# *Microbial Biotechnology* is 10!

**DOI:** 10.1111/1751-7915.12876

**Published:** 2017-10-24

**Authors:** Kenneth Timmis, Joan Timmis

**Affiliations:** ^1^ Institute of Microbiology Technical University Braunschweig Germany

This year, *Microbial Biotechnology* (MBT), which was created as a sister Journal of *Environmental Microbiology* for publication of the best research in applied microbiology, attained the age of 10 which, in journal lives, probably corresponds to mid‐puberty! Certainly, the Journal has recently been experiencing remarkable spurts in growth of submissions, which is healthy because it enables the Journal Editors, Editorial Board and other Reviewers, to be increasingly selective and the Journal to publish papers of increasing quality and originality.

Quality refers to both individual published works and to new or topical thematic collections that represent initiatives of the Editors, Editorial Board Members and author–readers of the Journal. As can be appreciated from the MBT website, MBT philosophy is not only to publish excellent author‐initiated submissions, but also to stimulate and support the field of microbial biotechnology, by soliciting papers on, and showcasing, (to become) hot topics or technical developments that will catalyse significant advances in the field. It does this with *Minireviews,* and *Thematic and Special Issues*. On a biannual basis, it publishes a *Crystal Ball* Feature in which invited luminaries from both inside and outside the field predict – sometimes wildly – which new ideas, technologies, discoveries or applications from other disciplines, will drive the most important advances in microbial biotechnology (see *Crystal Ball‐2017*: http://onlinelibrary.wiley.com/doi/10.1111/mbt2.2017.10.issue-1/issuetoc). In order to identify bottlenecks in the roads to important medium‐term biotechnology goals and encourage efforts to circumvent such obstacles, MBT recently published the feature *Microbial Biotechnology – 2020*, in which, leading researchers defined key goals for 2020 and developed road maps to attain these goals. Most recently, the previous issue of MBT was devoted to an assessment of the current and potential contributions of microbial biotechnology to attainment of the *Sustainability Development Goals* (SDGs) specified by the United Nations (*Special Issue: The contribution of microbial biotechnology to Sustainability Development Goals*; http://onlinelibrary.wiley.com/doi/10.1111/mbt2.2017.10.issue-5/issuetoc), with the goals of not only encouraging younger researchers to appreciate the wider socio‐political context in which their work plays out, but also exposing to policymakers the incredible range of important applications that characterizes work in microbial biotechnology, and their impacts on key issues and problems currently facing the planet and its inhabitants.

One of numerous global problems facing the biosphere is the accumulating mountain or, more accurately, sea of plastic waste, and the current issue is devoted to the potential of microbial biotechnology to contribute to mitigation strategies and to channel plastic waste into a value chain that includes environmentally friendly bioplastics and valuable synthons.

In its efforts to stimulate the field, MBT focuses considerable effort on young researchers and their careers. One initiative it started this year was to publish a series of *Editorials* that identify exciting and rapidly expanding sectors offering excellent employment opportunities for young people. The current sector emphasized is the microbiome (*Editorial Series: The microbiome as a source of new enterprises and job creation*) and has thus far featured *inter alia* microbiome transplants, microbiome applications in agriculture and microbiome‐based forensics. The series is ongoing, and further, highly interesting contributions are in the pipeline.

It is a truism that serendipity underpins many important discoveries (forgetting an agar plate in the cold room, only to find an interesting mutant when the plate is re‐discovered during a clean‐out). However, serendipitous discoveries tend to be made by researchers who are (critically) receptive to the unusual and unexpected, and to the possibilities that current laboratory lore may be incorrect or laboratory paths may be leading to dead ends, and/or are able to make connections between apparently unrelated topics. To both encourage a healthy critical attitude to laboratory lore and directions and to expose young people to important and interesting topics and advances that may not be on their radar screens, MBT publishes a series, *Microbiome Yarns*, that exploits humour and fantasy to draw attention to key papers in the fields featured. While some of the current initiatives will continue beyond 2017, other new ones will be launched, so watch this space!

Since its inception, MBT has benefitted from an outstanding team of dedicated Editors who devote much time and energy to provide authors with the best possible support and critical, objective advice. The Founding Editors were Willem de Vos, Willy Verstraete, Juan Luis Ramos, Marty Rosenberg and Kenneth Timmis, with Joan Timmis as Journal Administrator. After many years of dedicated service, Marty and Willy rotated off, to be replaced by Siegfried Vlaeminck and Auxi Prieto. Larry Wackett has been Web Alert Editor since the launch of the Journal, and Antoine Danchin joined recently as Genome Update Editor. It has been an incredible personal experience for us to work with such a cohesive, inspiring and fun group of scientists, and we take this opportunity to express our intense and undying gratitude for their exceptional dedication to the Journal and the field in general.

But, we should also like to single out Juan Luis Ramos for particular mention: although all Editors have substantial workloads, Juan Luis, who is also Minireview Editor and Special/Thematic Issue Editor, and hence responsible for the recruitment and editorial handling of practically all the minireviews published in the Journal (as well as in Environmental Microbiology and Environmental Microbiology Reports), and for the handling of the majority of SI/TI submissions, has an exceptional workload, far exceeding those of others, which he cheerfully and efficiently manages, on top of all the other demanding responsibilities that come with his day job. Thank you so much Juan Luis!

Of course, the task of the Editors can only be accomplished through the reviews we receive from our outstanding Editorial Board and other *ad hoc* reviewers, who represent the best talent in the field and who provide knowledgeable, constructive and supportive critiques, and decision recommendations, on manuscripts they review. Moreover, Editorial Board Members contribute significantly to the development of Journal policy, and some, in particular, Victor de Lorenzo, Sang Yup Lee and Jack Gilbert, play vital roles in many Journal initiatives. Thank you all for your super contributions to the success of MBT!

And, of course, MBT would neither exist nor have experienced the success it enjoys, if it were not for its publisher, Wiley, who has always done a sterling job of production, management and marketing: thank you everyone at Wiley for your constant support and professional handling of the Journal.

And finally, a big thanks to the Society for Applied Microbiology who welcomed MBT into its journal stable and has provided a tremendous amount of moral and microbiological support: thank you so much Phil and Lucy!

## Conflict of interest

None declared.

